# Interferon Family Cytokines in Obesity and Insulin Sensitivity

**DOI:** 10.3390/cells11244041

**Published:** 2022-12-14

**Authors:** Ling-Yu Huang, Chiao-Juno Chiu, Chung-Hsi Hsing, Yu-Hsiang Hsu

**Affiliations:** 1 Institute of Clinical Medicine, College of Medicine, National Cheng Kung University, Tainan 701, Taiwan; 2 Department of Medical Research, National Taiwan University Hospital, Taipei 100, Taiwan; 3 Department of Anesthesiology, Chi Mei Medical Center, Tainan 710, Taiwan; 4 Department of Medical Research, Chi Mei Medical Center, Tainan 710, Taiwan; 5 Clinical Medicine Research Center, National Cheng Kung University Hospital, College of Medicine, National Cheng Kung University, Tainan 704, Taiwan; 6 Antibody New Drug Research Center, National Cheng Kung University, Tainan 701, Taiwan

**Keywords:** obesity, interferon, insulin resistance

## Abstract

Obesity and its associated complications are global public health concerns. Metabolic disturbances and immune dysregulation cause adipose tissue stress and dysfunction in obese individuals. Immune cell accumulation in the adipose microenvironment is the main cause of insulin resistance and metabolic dysfunction. Infiltrated immune cells, adipocytes, and stromal cells are all involved in the production of proinflammatory cytokines and chemokines in adipose tissues and affect systemic homeostasis. Interferons (IFNs) are a large family of pleiotropic cytokines that play a pivotal role in host antiviral defenses. IFNs are critical immune modulators in response to pathogens, dead cells, and several inflammation-mediated diseases. Several studies have indicated that IFNs are involved in the pathogenesis of obesity. In this review, we discuss the roles of IFN family cytokines in the development of obesity-induced inflammation and insulin resistance.

## 1. Obesity and Metabolic Syndrome

Obesity has become a global epidemic disease. The prevalence of obesity has tripled globally since 1975 (https://www.who.int/, accessed on 9 June 2021). A body mass index (BMI, the weight in kilograms divided by the square of the height in meters) equal to or more than 30 is defined as obese, which is associated with increased morbidity and mortality [1]. Obesity is a major risk factor for chronic diseases including cardiovascular disease, diabetes mellitus (DM), chronic kidney disease, cancers, musculoskeletal disorders, and obstructive sleep apnea. More than 80% of obese individuals are prone to developing insulin resistance during their lifetime. Enlarged adipocytes release free fatty acids (FFAs), lipopolysaccharide, reactive oxygen species, and proinflammatory cytokines, causing lipotoxicity in nonadipose organs, including the liver, muscle, and pancreas. Dysregulated organelles contribute to systemic inflammation, which causes insulin resistance by inhibiting the insulin signaling pathways [2,3].

According to the National Cholesterol Education Program Adult Treatment Panel III (NCEP-ATP-III), there are several symptoms, including abdominal obesity, hypertension, dyslipidemia, and insulin resistance or overt type 2 DM, for the diagnosis of metabolic syndrome. Nonalcoholic fatty liver disease (NAFLD) is characterized by fat accumulation in the liver and can develop into more severe disease—hepatic steatosis and nonalcoholic steatohepatitis (NASH) [4,5]. However, there are some limitations to this disease nomenclature. Fatty liver diseases are also accompanied by metabolic syndrome and alcohol-associated or other chronic diseases. Therefore, a new term has been proposed: metabolic dysfunction-associated fatty liver disease (MAFLD). Genetic alterations in lipid metabolism in the liver, including increased de novo lipogenesis, increased mitochondrial fatty acid oxidation (FAO), increased uptake of FFAs, alterations in triglyceride (TG) secretion, insulin resistance in adipose tissues and skeletal muscle and gut microbiota, are involved in the pathogenesis of MAFLD [6]. Adipose tissue macrophages (ATMs) from obese mice induced hepatic neutrophil recruitment during NASH development [7]. Therefore, there is an urgent need to understand the crosstalk of inflammatory responses between adipocytes and liver cells.

Obesity, type 2 DM, and cardiovascular diseases are closely linked. The mechanisms underlying these pathogenic effects include adipokine dysregulation, inflammation, increased circulating FFAs, and altered energy storage causing atherosclerosis, endothelial cell dysfunction, and cardiometabolic disease [8]. In severe obesity, impaired insulin-stimulated glucose uptake, oxidative metabolism, and increased lactate production have been observed in skeletal muscle [9]. Skeletal muscle cells secrete cytokines that increase immune cell infiltration and promote skeletal muscle inflammation in obesity. IL-6, IL-8, IL-15, TNF-α, MCP-1 (CCL2) and other molecules, such as fibroblast growth factor 21, irisin, myonectin, and myostatin, are involved in obesity with insulin resistance or type 2 DM [10,11,12,13,14]. 

## 2. Crosstalk between Adipose Tissue and Immune Cells

Adipose tissue is composed of several types of cells, including adipocytes, preadipocytes, immature adipocyte precursors, endothelial cells, and immune cells. Three different types of adipocytes that exist in mammals are white adipocytes, brown adipocytes, and beige adipocytes. White adipocytes are the main source of storage of excess energy. In contrast, brown adipocytes can transduce energy into heat. When abnormal or excess energy is stored in the adipose tissue, the expansion of adipose tissue starts from either resident tissue precursors or existing adipocytes to form new (hyperplasia) or enlarged adipocytes (hypertrophy). Adipocytes act as endocrine regulators to modulate energy expenditure and systemic health. They secrete a variety of hormones, proteins, and peptides that are known as adipocyte-derived adipokines and participate in lipid metabolism, insulin sensitivity, blood pressure regulation, and inflammation [15]. For example, high levels of the proinflammatory markers C-reactive protein and IL-6 were found in obese people and represent risk factors for developing type 2 DM. Moreover, other adipokines, such as leptin, resistin, RBP4, and TNF-α, also have proinflammatory effects and influence cardiovascular functions [16]. 

Under homeostatic conditions, these adipose tissue-resident immune cells produce anti-inflammatory cytokines and maintain tissue homeostasis in nonobese individuals. Regulatory invariant natural killer T cells, γδT cells, type 2 innate lymphoid cells, eosinophils, regulatory T cells (Tregs), and M2-polarized ATMs are enriched in the adipose immune system. These cells produce IL-4, IL-5, IL-10, IL-13, and TGF-β to remodel adipose tissues and contribute to an anti-inflammatory and insulin-sensitive phenotype. 

In obese conditions, inflammatory immune cell accumulation in the adipose microenvironment is critical for causing insulin resistance and metabolic dysfunction. Macrophages were increased in obese individuals and participated in inflammatory pathways. Macrophage infiltration is increased in adipose tissues that switch to a proinflammatory microenvironment [17]. In obesity, adipocytes become hypertrophic and dysregulated, resulting in inflammatory changes in adipose tissue with the secretion of interferon-γ (IFN-γ) and TNF-α [18]. The adipose microenvironment is disturbed and enriched in M1-polarized ATMs, CD8^+^ T cells, type 1 T helper (Th1) cells, Th17 cells, and neutrophils [19,20]. Inflammatory cytokines or chemokines such as IL-6, IL-1β, IL-18, MCP-1, IL-8, and CXCL5 were higher in obese subjects and acted as mediators to activate or recruit immune cells. In addition, TNF-α, IFN-γ, and IL-1β are all associated with inflammation and insulin resistance [21]. Therefore, these cytokines or adipokines act as modulators that contribute to obesity-induced inflammation and metabolic diseases.

## 3. Interferons (IFNs)

IFNs are a family of cytokines that play critical roles in inflammation, immunoregulation, and tumor cell recognition [22]. They can be divided into type I, type II, and type III. Isaacs and Lindenmann discovered a substance that can protect cells from viral infections, IFN, in 1957 [23]. Type II and III IFNs were found in 1965 and 2003, respectively [24,25].

Type I IFNs are produced by plasmacytoid monocytes, dendritic cells (DCs), and macrophages after viral infection [26,27,28]. Type I IFNs can also be produced by almost every cell type, including leukocytes, fibroblasts, and endothelial cells [29]. Some studies have indicated that type I IFNs can be produced by bacterial stimulation. Mice were subjected to bacterial challenge with intranasal staphylococcal enterotoxin B, resulting in the expression of IFN regulatory factors (IRFs), including IRF1, IRF7, and IRF8, which elicited IFN responses [30,31]. These IFNs cooperate with other cytokines or chemokines to improve host antitumor effects and participate in immune responses [32,33]. High doses of type I IFNs exerted profound therapeutic effects in the murine B16 model through a direct antiangiogenic effect [34]. Further studies also showed that mice deficient in IFN α/β receptor (IFNAR) or signal transducer and activator of transcription (STAT) protein 1 failed to induce T-cell priming. IFNAR^−/−^ or STAT1^−/−^ mice had a defective ability to recruit CD8α^+^ DCs to tumors [35]. Another study also demonstrated that mice lacking IFNAR1 in DCs could not reject highly immunogenic tumor cells and that CD8α^+^ DCs could not cross-present antigens to CD8^+^ T cells [36]. The connection between type I IFN and autoimmune or inflammatory diseases is well known. For example, IFN was found in patients with systemic lupus erythematosus, rheumatoid arthritis, scleroderma, and Sjogren’s syndrome [37,38,39]. It also acts as a trigger for developing autoimmune thyroid disease [40].

In the human genome, thirteen functional type I IFN genes are located at 9p21.3, including IFN-α (IFNA1, IFNA2, IFNA4, IFNA5, IFNA6, IFNA7, IFNA8, IFNA10, IFNA13, IFNA14, IFNA16, IFNA17, and IFNA21), IFN-ω (IFNW1), IFN-ɛ (IFNE), IFN-κ (IFNK), IFN-ζ, IFN-τ (tau), IFN-δ and IFN-β (IFNB1), and 11 IFN pseudogenes [41,42]. They are recognized by IFNAR and activate Janus kinase 1 (JAK1) and tyrosine kinase 2 (TYK2). When activated, these two kinases phosphorylate the receptors and recruit STAT proteins. The IFN-stimulated gene factor 3 (ISGF3) complex is composed of STAT1, STAT2, and IRF9, translocates into the nucleus and binds to the promoter region of the IFN-stimulated response element (ISRE) to activate antiviral responses [43]. In addition, the STAT1 homodimer translocates into the nucleus and binds to gamma-activated sequences to induce proinflammatory genes, whereas the STAT3 homodimer indirectly inhibits inflammatory gene expression by being bound by the corepressor complex SIN3 transcription regulator homolog A, which is a repressor of inflammatory pathways [44]. These are the hallmarks of canonical pathways involved in type I IFN signaling. The noncanonical type I IFN signaling pathway is similarly activated by IFNs binding to IFNAR. The main noncanonical IFN pathways include the mitogen-activated protein kinase (MAPK) and phosphatidylinositol 3-kinase (PI3K)/mammalian target of rapamycin (mTOR) pathways [45].

Type II IFNs consist of only IFN-γ, which participates in innate and adaptive immune responses. For example, in early host immune responses, natural killer (NK) cells, CD4^+^ Th1 cells, and CD8^+^ T cells produce IFN-γ to improve antigen recognition in antigen-presenting cells such as macrophages and DCs, causing macrophage polarization to the M1 phenotype and increasing the levels of the proinflammatory cytokines IL-1β, IL-12, IL-23, and TNF-α [46].

Aberrant production of IFN-γ is also involved in autoimmune diseases [37]. NK cells and natural killer T (NKT) cells are the main producers of IFN-γ for innate immune responses [47]. When NK cells are stimulated to secrete IFN-γ, the pathway is receptor-mediated or cytokine-mediated. Activating receptors transmit signals into the cytoplasm through phosphorylation of immunoreceptor tyrosine-based activating motifs, resulting in activation of protein tyrosine kinases of the Src family and leading to activation of downstream signaling, including MAPK, PLC-γ, and the Son of sevenless/Ras pathways [48]. Cytokine-mediated NK cell activation is regulated by IL-12, which can enhance NK cell cytotoxic killing activity [49]. After infection, macrophages secrete IL-12, which interacts with its receptor on NK cells and then activates STAT4 and NF-κB to promote IFN-γ expression [50]. A previous study indicated that IL-15 acted in concert with IL-18 to stimulate IFN-γ production [51]. Another study also indicated that IL-2, which shares its β and γ chains with IL-15, produced IFN-γ in vitro [52].

NKT cells produce IFN-γ through the T-cell receptor (TCR) [53]. Some studies have indicated that macrophages, B cells, CD4^+^ T cells, and CD8^+^ T cells are involved in IFN-γ signaling to facilitate the immune elimination of tumors and effective immune responses [46,54,55,56,57]. IFN-γ production from CD4^+^ T cells or CD8^+^ T cells can be mediated by receptor- and cytokine-dependent mechanisms that are similar to NK cell stimulation. After the interaction between TCR and antigen, Src family tyrosine kinases are activated, leading to MAPK, Jun, and Fos activation that ultimately upregulates IFN-γ production [58]. In cytokine-dependent mechanisms, exposure to IL-12 and IL-18 can produce IFN-γ in CD4^+^ T cells or CD8^+^ T cells [59]. Signal transduction is dependent on IFN-γ interacting with the IFN-γ α/β receptor and activating the downstream JAK-STAT signaling pathway [60]. 

Type III IFNs include IFN-lambda 1 (IFN-λ1, also known as IL-29), IFN-λ2 (also known as IL-28A), IFN-λ3 (also known as IL-28B), and IFN-λ4 [25,61,62]. In humans, the IFN-λ locus is associated with hepatitis C virus infection [63]. Most classes of viruses and some bacterial products induce IFN-λ expression [25,61,64,65]. There are some cell types, including epithelial cells, hepatocytes, B cells, plasmacytoid DCs (pDCs), and macrophages, that can express functional IFN-λ receptors and respond to IFN-λ [66,67,68,69,70,71,72,73]. IFN-λs are found in innate immune defenses at mucosal barriers. In intestinal epithelial cells and airway epithelial cells, IFN-λ mediates antiviral protection to block viral replication and transmission [74,75,76,77,78]. In addition, Toll-like receptor (TLR) agonists protected vaginal epithelial cells from herpes simplex virus type 2 infection, and this mechanism is dependent on IFN-λ [79]. Furthermore, after virus infection in the upper airway, IFN-λ also regulates adaptive immunity. IFN-λ acts on CD103^+^ DCs and triggers thymic stromal lymphopoietin expression, thereby greatly enhancing the production of virus-specific CD8^+^ T cells and antibodies, resulting in improved resistance to infection with influenza viruses [80].

All type III IFNs signal through IFN-λ receptor 1 (also termed IL-28R1) and IL-10 receptor subunit-β (IL-10R2), which leads to JAK1 and TYK2 activation, resulting in STAT1–STAT2–IRF9 complex (which is known as the ISGF3 complex) translocation into the nucleus and binding to IFN-stimulated response elements in the promoter regions of ISGs [81].

## 4. IFN Expression in the Development of Obesity

IFNs are not only associated with host defense but are also related to obesity. Compared with nonobese subjects, peripheral blood mononuclear cells (PBMCs) from obese subjects showed decreased IFN-α and IFN-β production after stimulation with TLR ligands [82]. To investigate whether the level of IFN-β is associated with metabolically unhealthy abdominal obesity (MUAO) due to obesity or metabolic state, we examined the correlation between the serum levels of lipoprotein binding protein and IFN-β in 65 abdominally obese subjects with a waist circumference ≥ 95 cm. However, there was no difference in these two parameters between the MUAO and metabolically healthy abdominally obese (MHAO) groups. This suggests that serum IFN-β and abdominal obesity are more closely related [83]. Similarly, waist circumference significantly correlated with CD3 and IFN-γ mRNA expression in type 2 DM patients, suggesting an association between insulin resistance and lymphocyte infiltration in adipose tissue [84]. A recent study indicated that insulin peptide-specific IFN-γ-related immune responses play a key role in ketosis-prone type 2 DM [85]. High-fat diet (HFD) feeding in a murine model of obesity-related insulin resistance and NAFLD increased the number of pathogenic intrahepatic CD8^+^ T cells. Diet-induced obesity (DIO) promoted intrahepatic type I IFN responses activating T-cell pathogenicity, which resulted in NAFLD progression and glucose dysregulation [86].

HFD-induced mouse models can also activate the cGAS-cGAMP-STING pathway. This pathway not only mediates type I IFN responses but also contributes to increased insulin resistance and the development of NAFLD [87]. In addition, a previous study showed that influenza infection in obese mice caused higher mortality rates due to impaired induction of both type I and type III IFN responses [88]. Moreover, COVID-19 infection of obese individuals also resulted in delayed and attenuated type I and III IFN responses [89].

The overexpression of proinflammatory cytokines in obesity is associated not only with local immune responses in visceral adipose tissues (VAT) but also with chronic systemic inflammation. A study demonstrated that serum levels of IFN-γ from obese subjects were significantly related to general obesity (determined by BMI) and central obesity (determined by waist-hip ratio) [90]. Similarly, higher levels of IFN-γ were found in adipose tissue from obese animals [21]. However, one study showed that prenatal dexamethasone and postnatal HFD decreased IFN-γ production and caused aberrant site-specific and age-dependent histone modification in male Sprague Dawley rat offspring [91]. In obese children, increased production of IFN-γ secreted from CD4^+^ T cells was associated with insulin resistance and NASH [92]. A higher percentage of CD4^+^ and CD8^+^ T cells in VAT was observed in DIO mice than in lean mice. Moreover, T cells secreted more IFN-γ in obese adipose tissues. Greater IFN-γ secretion from PMA/ionophore-stimulated epididymal adipose tissue was also observed at week 6 and week 12 in HFD-treated C57BL/6 mice [21,93]. 

**Figure 1:**
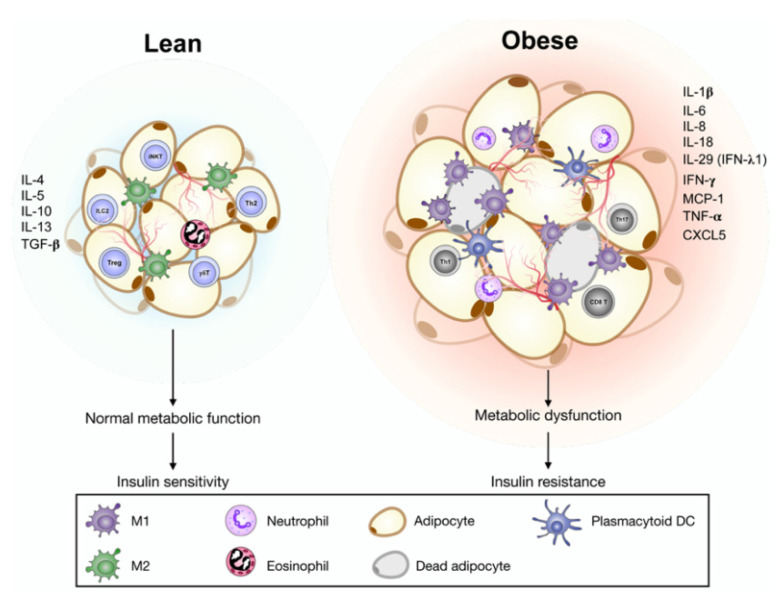
Adipocytes and immune cells act cooperatively to produce cytokines, chemokines, and interferons, contributing to obesity-related inflammation and metabolic dysregulation in human adipose tissue.

Fat accumulation in the liver can lead to the development of more severe hepatic steatosis and NASH from NAFLD. A study showed that IFN-λ3 levels were associated with the development of NASH in SD rats [94]. Another study demonstrated that serum IFN-λ1 levels were higher in obese patients than in healthy individuals. IFN-λ1 was expressed in VAT isolated from obese patients [95,96]. We summarized the association between the IFN family and obesity as well as insulin sensitivity (Figure 1).

## 5. IFN Expression in the Regulation of Insulin Sensitivity

A previous study demonstrated that long-term incubation of IFN-α at low concentrations increases the high-affinity binding of insulin and modulates insulin sensitivity and glucose transport in human adipocytes [97]. In contrast, long-term HFD feeding reduced the numbers of Tregs in VAT and elevated levels of IFN-α, resulting in insulin resistance [98]. After type I and type II IFNs bind to their receptors to activate STAT through the JAK family, cytoplasmic STAT translocates to the nucleus and promotes the transcription of SOCS [99]. Increased SOCS1 expression has been observed in the liver, muscle, and adipose tissues of obese db/db mice. Moreover, SOCS is associated not only with the JAK-STAT pathway but also with insulin signaling [100]. For example, IFN-β induces SOCS1 expression, which suppresses insulin-induced tyrosine phosphorylation of insulin receptor substrate 1 (IRS-1). IFN-γ stimulates SOCS3 expression, which results in the degradation of IRS-1. In addition, IFN-β-induced SOCS1 expression and IFN-γ-induced SOCS3 expression are mediated by STAT1 and STAT3. Moreover, IFN-β and IFN-γ induce SOCS isoforms leading to insulin resistance in 3T3-L1 adipocytes [101]. IRF7 is a major regulator of type I IFN-dependent immune responses [102]. A previous study indicated that increased levels of IRF7 were found in white adipose tissue (WAT), liver tissue, and gastrocnemius muscle of HFD-treated mice and ob/ob mice. Another study also indicated that induction of IFN-α from pDCs induced more transcription of ISG in VAT, which is positively associated with adipose tissue and systemic insulin resistance [103]. 

The causal relationship between IFN-α and insulin resistance was elucidated in healthy individuals treated with natural human leukocytes IFN-α by impairing glucose tolerance and insulin sensitivity [104]. In addition, type I IFNs were found to play a vital role in the diabetic microenvironment by impairing insulin secretion and mitochondrial bioenergetics and enhancing autoimmune attacks on pancreatic β cells [105]. Another study also showed that IFN-α treatment in human islets and human EndoC-βH1 cells triggered endoplasmic reticulum stress, decreased insulin production and altered the functional state of β cells [106]. Some studies have been conducted to investigate the association between insulin resistance and IFN-γ. For example, there was an association between IFN-γ, insulin resistance, and MetS in obese children [92,107]. In HFD mice, hyperglycemia with a reduction in adiponectin increased T-cell glycolysis, resulting in increased IFN-γ and IL-17 and insulin resistance [108]. Ablation of NLR family pyrin domain containing 3 (NLRP3) inflammasomes in obesity reduced inflammation and adipose tissue IFN-γ and improved insulin sensitivity [109]. 

IFN-γ-induced protein 10 (IP-10) is a proinflammatory chemokine. The level of IP-10 was higher in NAFLD patients and in those with incident diabetes. Moreover, IP-10 levels were associated with MCP-1, TNF-α, and insulin resistance. Therefore, IP-10 can be an independent predictor of liver injury, incident diabetes and insulin resistance [110]. One study showed that TNF-α and IFN-γ could also induce nitric oxide formation, leading to partially impaired glucose-stimulated insulin secretion and causing DNA damage [111]. In addition, one study showed an association between insulin-dependent diabetes mellitus (IDDM) and the expression of IFN-γ in pancreatic β cells. IFN-γ transgenic mice suffered inflammatory destruction of the islets [112]. IFN-γ expression in insulitis lesions was reduced in insulin immunization of nonobese diabetic (NOD) mice [113]. According to the abovementioned findings, IFN-α could be mediated by immune cells and induce ISG transcription during HFD feeding. However, the mechanism by which local pDCs regulate IFN-α is still under investigation. Moreover, the mechanism by which IFN-β and IFN-γ induce SOCS isoforms needs to be further investigated, which is a potential target to inhibit or prevent SOCS signaling. The effects of IFN-γ on insulin sensitivity are dependent on the stimulation of SOCS3 expression or the induction of nitric oxide formation and should be further confirmed.

## 6. Impact of IFNs on AT Metabolism and Remodeling during Obesity

Glyceroneogenesis is a metabolic pathway that synthesizes glycerol 3-phosphate from noncarbohydrate precursors. The key enzyme that regulates this metabolic pathway is the cytosolic isoform of phosphoenolpyruvate carboxykinase (PEPCK-C), which is considered a regulator of fatty acid efflux. In addition, one study demonstrated that PEPCK-C decreased in adipose tissues in IFN-γ-treated mice. IFN-γ-induced overproduction of fatty acids resulted in increased plasma fatty acids, which may correspond to the onset of insulin resistance [114]. Moreover, the production of IFN-γ is dominated by CD4^+^ T cells, which are prone to Th1 cytokine profiles and related to leptin, insulin resistance, and NASH [92]. Another study also suggested that pathogenic IFN-γ-secreting CD4^+^ Th1 cells accumulated in the VAT of DIO mice, overwhelming the high proportion of CD4^+^Foxp3^+^ T cells. Therefore, pathological T cells are involved in worsening metabolic regulation. Proinflammatory T-cell-derived IFN-γ was also found to induce insulin resistance, downregulate the levels of adipogenic genes and reduce lipid storage in human Simpson–Golabi–Behmel syndrome (SGBS) cells through the JAK-STAT pathway. These findings suggested that IFN-γ played a pivotal role in mediating T-cell activation in obesity and insulin resistance via the JAK-STAT pathway [115]. Furthermore, 3T3-L1 adipocytes could present endogenous ligands to NKT cells through CD1d, which leads to IFN-γ production. Depletion of adipocyte-specific CD1d reduced VAT mass and enhanced insulin sensitivity in DIO mice. Therefore, the interactions between NKT cells and CD1d-expressing adipocytes may play important roles in regulating adipose tissue metabolism [116]. 

IFN-γ released from omental adipose tissues obtained from obese individuals altered the adipocyte phenotype, which is characterized by fewer lipid droplets of increased size, impaired insulin sensitivity, and adiponectin release [117]. Moreover, IFN has been found to exert inhibitory effects on the differentiation of mouse 3T3-L1 adipocytes and decrease de novo lipid biosynthesis [118]. Virus-derived IFN-γ can also downregulate insulin receptor expression in skeletal muscle, causing systemic insulin resistance and promoting compensatory hyperinsulinemia to induce antiviral CD8^+^ T-cell responses in humans and mice, which suggests a circulating loop between the endocrine system and immune system [119]. In addition, pDCs, the main producers of type I IFNs, are among the immune cell populations involved in innate and adaptive immune responses. In lean individuals, Treg cells regulate local and systemic inflammation and metabolism in adipose tissues. Two studies indicated that Tregs were reduced and pDCs were recruited to the adipose tissue, which signaled through type I IFN signaling or secreted IFN-α, causing insulin resistance in DIO mice. This result suggested that blocking the pDC-IFN-α axis could restore insulin sensitivity and Tregs in VAT [98,120]. Therefore, IFN-γ could be induced by CD4^+^ T cells and the NKT/CD1d cell axis, leading to more pathogenic T cells and overproduction of fatty acids. However, how and why these immune responses trigger IFN-γ production and affect local and systemic metabolic responses still need to be investigated.

Glycolysis is the pathway that generates pyruvate/lactate after glucose uptake, which regulates insulin secretion and the metabolic functions of cells. Previous studies have shown that type I IFNs produced by pDCs induce an increase in FAO and glycolysis [121]. Furthermore, IFN-β treatment in adipocytes enhanced the expression of glycolysis-associated enzymes and promoted the basal extracellular acidification rate and oxygen consumption rate [122]. IFN-β also regulates metabolic events that are important for the induction of a rapid antiviral response [123]. Taken together, the type I IFN axis not only alters adipocyte metabolism but also enhances antiviral responses by modulating glucose metabolism. Therefore, therapeutic induction of type I IFN selectively in ATMs may provide a novel approach for treating obesity.

**Figure 2:**
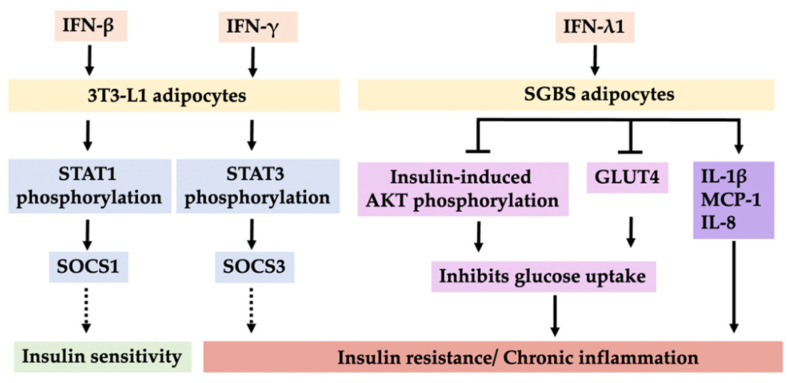
Schematic model of different mechanisms of insulin sensitivity and resistance by IFNs in 3T3-L1 adipocytes and SGBS adipocytes.

WAT expansion and remodeling are risk factors for developing metabolic syndrome in obesity. One study showed that IFN-β1 treatment restored insulin sensitivity and improved glucose homeostasis in the DIO mouse model. In addition, IFN-β1 blocked adipose tissue expansion and body weight gain and was linked to increased adipose tissue thermogenesis [124]. Therefore, these findings reveal that this immune-modulating IFN-β1 treatment can attenuate adipose tissue inflammation and protect against obesity and its related complications. Although IFN-λ1 has been found to play a critical role in host defense against microbes and virus infection, one study showed that IFN-λ1 downregulated adipogenic markers, including peroxisome proliferator-activated receptor-γ (PPAR-γ), FABP-4, and lipoprotein lipase, in mature SGBS adipocytes. Moreover, IFN-λ1 decreased glucose uptake and insulin sensitivity in SGBS adipocytes by reducing glucose transporter 4 (GLUT4) and phosphorylation of protein kinase B (AKT) signals. In vivo, IFN-λ1 treatment also worsened glucose homeostasis and insulin sensitivity as determined through glucose and insulin tolerance tests (ITTs) in HFD mice [95]. We summarized the signal pathways of the IFNs in 3T3-L1 adipocytes and SGBS adipocytes (Figure 2).

## 7. Proposed Mechanisms of IFNs in Obesity-Associated Inflammation

In excess nutrient conditions, adipose tissues release inflammatory mediators, including TNF-α and IL-6, decrease adiponectin expression, and recruit and activate immune cells in adipose tissues, resulting in a proinflammatory state and oxidative stress. People with obesity have been associated with impaired immune responses, leading to increased susceptibility to infection and mortality. Type I IFNs have been found to be involved in early immune responses after viral infections and are regulated by SOCS1 and SOCS3 [23,125]. For example, COVID-19 patients had high levels of proinflammatory cytokines resulting in the induction of SOCS1 and SOCS3, impairing type I and III IFN responses [89]. A study showed that obesity contributed to impaired IFN-α and IFN-β responses, which made people respond inadequately to viral infections [82]. Another study also indicated that impaired IFN-α, IFN-β, and IFN-λ were caused by attenuated IRF7 in influenza infection of obese mice [88]. These findings have drawn more attention to the effects of IFN production and IRF7 in obese individuals and individuals with other metabolic disorders. IRF7 mediates obesity-associated MCP-1 transcription in 3T3-L1 adipocytes. IRF7^−/−^ mice had lower MCP-1 expression in epididymal WAT during HFD feeding [126]. 

The cGAS-cGAMP-STING pathway has been described as a cytosolic DNA sensor to detect pathogen-derived DNA and activate type I IFN responses. A study demonstrated that obesity induced mtDNA release and activated the cGAS-cGAMP-STING pathway, resulting in chronic inflammation in adipose tissues. In addition, the findings revealed that the lack of DsbA-L, a chaperone-like protein originally identified in the mitochondrial matrix, greatly increased the mRNA expression of IFN-α and inflammatory genes such as TNF-α, IL-18, and MCP-1. Therefore, overexpression of DsbA-L can prevent chronic inflammation, insulin resistance, and metabolic dysfunction resulting from mtDNA-induced activation of the cGAS-cGAMP-STING signaling pathway. Targeting the cGAS-cGAMP-STING pathway in adipose tissue may provide another approach to ameliorate obesity-associated inflammation and metabolic disorders by downregulating the levels of proinflammatory genes, including IFN-α [127]. 

In obesity, increased FFAs cause metabolic stress, which induces endothelial inflammation and dysfunction [128]. Increasing evidence has shown that STING plays a critical role in immunity and inflammation. After activation, STING recruits TANK-binding kinase 1 and IRF3, resulting in IRF3 phosphorylation and activation. Then, IRF3 dimerizes and translocates into the nucleus, promoting the transcription of inflammatory factors such as IFNs [129]. A study suggested that palmitic acid, one type of FFA involved in obesity, induced the activation of the STING-IRF3 pathway and the expression of inflammatory proteins, including VCAM-1, MCP-1, IFN-γ, IL-1, and ICAM-1. In addition, STING activation in adipose tissues from DIO mouse models is involved in adipose tissue inflammation and insulin resistance. Therefore, targeting STING signaling not only has an impact on cardiovascular disease, but also on metabolic syndrome in obese individuals [130]. 

Type I IFNs are mediators that orchestrate both innate and adaptive immune responses. The type I IFN/IFNAR axis regulates inflammatory vigor in both adipocytes and myeloid cells and contributes to obesity-associated pathogenesis in mice. Therefore, targeting the type I IFN/IFNAR axis may be a possible intervention in dampening obesity-driven metabolic derangements [122]. A previous study showed that overexpression of IFN-β1, an immune-modulating cytokine, attenuated HFD-induced adipose inflammation and improved insulin sensitivity and glucose homeostasis [124]. Therefore, immune regulation in obesity-associated adipose inflammation may protect against obesity and its related pathologies.

In obese children, CD4^+^ T cells secreted more IFN-γ and were prone to shift to the Th1 phenotype [92]. Accumulating evidence indicates that increased T cells in adipose tissues are among the main causes of obesity-related metabolic syndrome. Adipose-resident T cells (ARTs) from DIO mice had a high frequency of IFN-γ expression and increased inflammatory mediators. In addition, the elimination of ARTs in visceral fat enhanced insulin sensitivity during early-stage obesity and reduced the epididymal adipose tissue expression of T-cell-derived IFN-γ [131]. Development of T-cell depletion specific to visceral fat may help reduce comorbidities of obesity. In addition, a study showed that T-cell priming in epididymal adipose tissues induced IFN-γ production in response to PMA/ionophore stimulation by week 6 of HFD feeding. IFN-γ release is accompanied by CD11c^+^ ATM recruitment, leading to inflammation and insulin resistance [93]. Moreover, 3T3-L1 adipocytes could present endogenous ligands to NKT cells, which promoted IFN-γ production, resulting in enhanced CD1d and CCL2 expression by stimulated adipocytes. Additionally, adipocyte-specific CD1d deletion inhibited the infiltration of M1 macrophages into adipose tissues [116]. Adipocytic 3T3-L1 cells stimulated with murine recombinant IFN-γ increased the levels of the T-cell chemoattractants IP-10, MIG, CC and CXC chemokine families, IFN-inducible T-cell α chemoattractants, MCP-1, and MCP-2. Moreover, media from IFN-γ-treated adipose tissues included higher levels of TNF-α. These results demonstrated that IFN-γ could regulate inflammatory gene expression specifically in adipose tissues [21]. Similarly, treatment of SGBS preadipocytes with IFN-γ significantly increased the release of MCP-1 [84]. 

To compare the difference between VAT and subcutaneous adipose tissue, stromovascular cell fractions were extracted from obese individuals. The results showed that IFN-γ-expressing NK cells were increased in VAT compared with subcutaneous adipose tissue. NK cell-derived IFN-γ regulated the macrophage phenotype in human VAT: in other words, IFN-γ upregulated the expression of TNF-α, an M1 cytokine, and downregulated the expression of IL-10, an M2 cytokine. These findings suggest that NK cells and IFN-γ regulate ATM-mediated inflammation in human obesity [18]. Chronic inflammation during obesity is regarded as a contributor to diseases such as type 2 DM, atherosclerosis, and certain cancers. Innate immune cells can recognize pathogen-associated molecular patterns through the expression of pattern-recognition receptors such as TLRs and NLRs. Among the NLR family members, the NLRP3 inflammasome can recognize nonmicrobial origin danger signals and activate caspase-1 as well as IL-1β and IL-18 secretion. In obese type 2 DM patients, NLRP3 expression is reduced in adipose tissues along with decreased inflammation and improved insulin sensitivity. Moreover, ablation of the NLRP3 inflammasome lowered the expression of IL-18 and IFN-γ and macrophage-T-cell interactions during obesity [109]. 

## 8. Modulation of IFNs in Animal Models of Obesity

According to the abovementioned findings, IFN-α, IFN-β, IFN-γ, and IFN-λ1 have been associated not only with immune responses during infection but also with obesity and its related metabolic syndrome. Therefore, transgenic animal models have been generated to study the underlying mechanisms of obesity development. There were higher numbers of CD4^+^ and CD8^+^ T cells in VAT in DIO obese C57BL/6 mice. Moreover, T cells from obese adipose tissues produced more IFN-γ than controls. IFN-γ^−/−^ mice fed a HFD improved insulin sensitivity, reduced the expression of MCP-1 and STAT-1, reduced leptin and cholesterol levels, and limited inflammatory cell infiltration and accumulation. However, IFN-γ participates in innate and adaptive immune responses and improves antigen recognition in antigen-presenting cells. Conditional ablation of IFN-γ in immune cells such as T cells requires further investigation [21]. Another study indicated that reduced body weight was associated with reduced food intake, increased physical activity, decreased NPY and AgRP (suppressed hunger-stimulating neuropeptides), and increased POMC (appetite-suppressing neuropeptide) in IFN-γ^−/−^ C57BL/6J mice compared with control mice fed a standard chow diet. In addition, IFN-γ^−/−^ mice had lower blood glucose levels during the ITT, suggesting that improved glucose tolerance was associated with increased insulin sensitivity. Increased whole-body insulin sensitivity and glucose tolerance resulted from decreased G6Pase activity associated with hepatic insulin sensitivity. Therefore, IFN-γ plays a crucial role in regulating energy balance [132]. 

Stromal vascular fraction (SVF) is a dominant inflammatory component of adipose tissues. SVF from obese WT mice and obese IFN-γ^−/−^ mice showed that the expression of TNF-α was increased, while IL-10 was decreased in response to IFN-γ treatment. Compared with obese WT mice, ATM from obese IFN-γ^−/−^ mice exhibited reduced TNF-α expression, and the relative frequency of CD11c^+^ cells was decreased, which has been shown to designate an obesity-specific M1 inflammatory ATM subpopulation. Moreover, the numbers of NK cells were decreased in VAT from obese IFN-γ^−/−^ mice compared to obese WT mice. Insulin sensitivity was modestly increased, and adipocyte size was modestly decreased in obese IFN-γ^−/−^ mice. According to these results, IFN-γ could regulate cytokine expression and predispose SVF cells toward M2-ATM. In addition, HFD-fed IFNAR^−/−^ mice were protected against diet-induced weight gain compared with B6 HFD control mice, which was associated with smaller subcutaneous fat depots and lower total fat mass. Furthermore, after 18 weeks of HFD feeding, the accumulation of proinflammatory macrophages in VAT was decreased in IFNAR^−/−^ mice. Another study also demonstrated that obese IFNAR^−/−^ mice exhibited improved glucose metabolism, based on improving glucose tolerance tests (GTTs) and ITTs and reduced hepatic CD4^+^ T cells. AdipoqcreIFNAR^fl/fl^ mice that specifically depleted IFNAR expression in adipocytes and littermate controls (Cre−IFNAR^fl/fl^) fed a long-term HFD exhibited improved glucose metabolism without influencing liver TG levels and hepatocellular damage [122]. 

IFNαR1-deficient (IFNαR1^−/−^) mice fed a HFD showed reduced liver weight, TG content, and lipid content compared to WT HFD-fed controls. Moreover, IFNαR1^−/−^ mice prevented DIO-induced insulin resistance by improving GTTs, ITTs, and pyruvate tolerance tests and reducing plasma fasting glucose and insulin levels. However, these effects were dependent on HFD feeding, while NCD-fed IFNαR1^−/−^ mice displayed no differences in metabolic tests. HFD-fed IFNαR1^−/−^ mice also exhibited lower levels of intrahepatic CD8^+^ and CD4^+^ T cells, Ki67^+^CD8^+^ and Ki67^+^CD4^+^ T cells, and TNF-α^+^CD8^+^ T cells. Overall, these findings indicated that type I IFN promoted the proliferation and activation of intrahepatic T cells, resulting in proinflammatory cytokine expression during DIO, and was involved in the pathology of metabolic diseases, including insulin resistance [86]. On the other hand, IFNαR1^−/−^ mice exhibited resistance to weight gain and were dependent on type I IFN responses originating in macrophages, which indicates that the selective induction of type I IFN in ATMs may provide a new strategy for treating obesity [133]. Although IFN-γ^−/−^ or IFNαR1^−/−^ mice displayed metabolic improvement and decreased proinflammatory responses, the impact of IFN ablation must be considered due to increased susceptibility to infection.

There are nine IRFs (IRF1-IRF9) in mammals. IRFs can regulate type I IFN expression. IRF1^−/−^ mice have been found to suppress spontaneous insulitis and DM in NOD mouse models by increasing CD4^+^ and Mac-1^+^ splenic cells and decreasing CD3^+^ and CD8^+^ cells and the IFN-γ/IL-10 ratio. Although IRF3 expression was higher in HFD-treated mice, IRF3 overexpression improved the HFD-induced dysfunction of insulin sensitivity. IRF3^−/−^ mice exhibited hepatic steatosis and insulin resistance. Therefore, IRF3 may be a promising target for treating metabolic diseases. IRF4 has been found to regulate macrophage polarization in adipose tissues during HFD-induced obesity. Deletion of IRF4 in macrophages increased proinflammatory cytokines and impaired insulin sensitivity in cocultured adipocytes. Global or myeloid cell-specific IRF5 deficiency improved insulin sensitivity, remodeled adipose tissue and recruited alternatively activated macrophages [134]. IRF9 expression was lower in livers from both DIO and genetically (ob/ob) obese mice. After consuming an HFD, IRF9^−/−^ mice were more obese and displayed impaired insulin sensitivity and glucose tolerance versus WT controls. In addition, the levels of tyrosine phosphorylation of IRS1 and serine phosphorylation of AKT were lower in the livers of IRF9^−/−^ mice than in those of WT mice. TG, total cholesterol, low-density lipoprotein, FFA, and β-hydroxybutyrate levels were higher in the sera of IRF9^−/−^ mice, while the level of high-density lipoprotein was lower. Serum levels of leptin and resistin were also higher and adiponectin was also lower in IRF9^−/−^ mice compared to WT controls. These findings suggest that IRF9 is involved in immune responses and metabolic regulation. Liver-specific IRF9 overexpression ameliorated these phenotypes in both DIO and genetically (ob/ob) obese mice. Importantly, IRF9 could upregulate the expression of PPAR-α target genes. Hepatic PPAR-α overexpression rescued obesity-induced metabolic disorders and inflammation in IRF9^−/−^ mice. These results shed light on the role of IRF9 in metabolic dysfunction [135]. Meanwhile, another study showed that IRF7^−/−^ mice gained less weight and had improved glucose and lipid homeostasis as well as insulin sensitivity. In addition, IRF7^−/−^ mice exhibited ameliorated diet-induced hepatic steatosis and decreased local and systemic inflammation. Therefore, IRF7 is believed to be involved in energy metabolism and insulin sensitivity [136]. However, another study indicated that IRFs play versatile roles in the pathogenesis of type 1 DM by affecting β cells, immune cells such as DCs, T cells and macrophages and type I and II IFN [137]. In summary, IRFs are a double-edged sword that can be identified as targets for both pathogenic and protective mechanisms in metabolic diseases.

## 9. Therapeutic Perspective of IFN in Obesity and Insulin Sensitivity

Cytokines are among the key regulators in mediating normal immune responses, representing a double-edged sword that is involved in the pathology of several diseases, but can also be used as a treatment for metabolic diseases [138,139]. Thus, there is a critical need to identify the actions of cytokines for novel therapeutic candidates. IFN-α is produced in response to viral infection and is a therapeutic target in some cancers and viral infections. Human IFN-α A/D treatment reduced body weight and adipocyte cell size in DIO C57BL/6 mice. In addition, IFN-α A/D treatment increased the number of apoptotic cells in 3T3-F422A adipocytes. Therefore, IFN-α therapy may affect weight change partly by increasing apoptosis of adipocytes [140]. IFN-α plays a key role in patients with type 1 DM. Targeting IFN-α or its receptor or pDCs may provide a potential therapeutic strategy against the development of type 1 DM. For example, one study showed that blocking IFN-α signaling prevented β cell death in mice [141]. Overexpression of IFN-β1 in HFD-treated mice resulted in less immune cell infiltration and lower inflammatory responses in adipose tissue. IFN-β1 could also systemically prevent adipose tissue expansion and body weight gain without impacting food intake. Moreover, IFN-β1 could improve insulin sensitivity and maintain glucose homeostasis. Consequently, IFN-β1 may be used as a potential target in immune-driven diseases [124]. A previous study also indicated that IFN-α-2b treatment could attenuate obesity development by decreasing body weight and improving dyslipidemia, which involved FAO and cholesterol decrement [142]. IFN-τ exhibits anti-inflammatory effects and low cell cytotoxicity even in high-dose treatment. In the DIO mouse model, IFN-τ treatment increased insulin sensitivity, which was accompanied by reduced proinflammatory cytokines and increased anti-inflammatory macrophages. Furthermore, IFN-τ could regulate macrophage polarization, cytokine profiles, and insulin signaling. As a result, IFN-τ may have the potential to provide a new therapeutic strategy for inhibiting the development of obesity and insulin resistance [143]. Cyclophosphamide (CYP)-induced NOD/Wehi mice treated with anti-IFN-γ antibody exhibited decreased incidence of diabetes and severity of insulitis. This suggests that IFN-γ may participate in the pathogenesis of IDDM [144]. A study demonstrated that type I IFN-based pre-BM transplant conditioning was applicable to the treatment of congenital metabolic disorder-Sly syndrome [145]. IFN-λ1 has been demonstrated to play an important role in regulating metabolic disease. IFN-λ1 expression in adipose tissue was higher in obese individuals. In addition, IFN-λ1 promoted inflammation and insulin resistance in both SGBS adipocytes and HFD-induced obese mice [95,96]. Consequently, these findings may provide new insights for IFN-λ1 targeting in treating obesity-induced inflammation and insulin resistance. The administration of different IFNs affected obesity-associated systemic insulin resistance and tissue inflammation (Table 1). Although overexpression of IFN-β1 or IFN-α A/D, IFN-α-2b, and IFN-τ treatment in HFD-treated mice resulted in body weight change, improvement of glucose metabolism, and reduced inflammatory responses, the side effects can vary between individuals. Furthermore, the dose, duration and timing of IFN treatment need to be further investigated.

## 10. Conclusions

Obesity is associated with immunity and is accompanied by imbalanced immune responses resulting from activated proinflammatory cells and cytokines. After the adipose microenvironment is perturbed, systemic homeostasis cannot be maintained, which is a risk factor for the development of insulin resistance. We summarized the association between the IFN family and obesity as well as insulin sensitivity. First, IFN-α, IFN-β, IFN-γ, and IFN-λ1 were associated with obesity and insulin sensitivity. Second, the type I IFN/IFNAR axis was found to regulate inflammatory vigor in adipocytes and contribute to obesity-associated pathogenesis in mice. Type I IFNs produced from pDCs induced increases in FAO and glycolysis. The pDC-IFN-α axis could restore insulin sensitivity and Tregs in VAT. IFN-γ can mediate the overproduction of fatty acids, T-cell activation, CD11c^+^ ATM recruitment, and inflammatory gene expression to impair insulin resistance. IFN-λ1 was higher in obese individuals, downregulated adipogenic markers, decreased glucose uptake, and reduced insulin sensitivity. Third, IFN-γ^−/−^ mice, IFNAR^−/−^ mice, IRF deficiency mice (IRF1^−/−^, IRF3^−/−^, IRF5^−/−^, IRF7^−/−^, and IRF9^−/−^) validated the crucial roles of IFN and IRF in metabolism and inflammation in adipose tissues. Finally, human IFN-α A/D, IFN-α-2b, IFN-τ treatment, and overexpression of IFN-β1 in DIO mice could reduce body weight, glucose levels, and insulin sensitivity and support anti-inflammation. Therefore, the use of prescription medications to treat obesity and metabolic diseases, such as metformin in combination with IFNs, may cause a synergistic effect. This review will guide future research regarding the IFN family in obesity and insulin sensitivity.

## Figures and Tables

**Table 1:** The effects of interferon treatment in obese mice.

Treatment					
	IFN-α A/D	IFN-α-2b	IFN-β1	IFN-τ	IFN-λ1
Treatment route	SC	IP	IV	Oral	IP
Mouse strain	B6	B6/Male	B6/Male	B6/Male	B6/Male
Body weight change	↓	↓	↓	−	↑
Adipocyte size	↓	−	−	−	−
FA oxidation	−	↑	−	−	−
Glucose homeostasis	−	↑	↑	−	↓
Inflammatory cytokine	−	↓	↓	↓	↑
Macrophage				M1 ↓ M2 ↑	M1/M2 ratio ↑
Insulin sensitivity	−	−	↑	↑	↓
Reference	[140]	[142]	[124]	[143]	[95]

Abbreviations: FA, fatty acid; M1, type I macrophages; M2, type II macrophages; SC, subcutaneous; IP, intraperitoneal.

## Data Availability

Not applicable.

## References

[1] Smith K.B., Smith M.S. (2016). Obesity Statistics. Prim. Care.

[2] Ahmed B., Sultana R., Greene M.W. (2021). Adipose tissue and insulin resistance in obese. Biomed. Pharmacother..

[3] James D.E., Stockli J., Birnbaum M.J. (2021). The aetiology and molecular landscape of insulin resistance. Nat. Rev. Mol. Cell Biol..

[4] Farrell G.C., Larter C.Z. (2006). Nonalcoholic fatty liver disease: From steatosis to cirrhosis. Hepatology.

[5] Parthasarathy G., Revelo X., Malhi H. (2020). Pathogenesis of Nonalcoholic Steatohepatitis: An Overview. Hepatol. Commun..

[6] Sakurai Y., Kubota N., Yamauchi T., Kadowaki T. (2021). Role of Insulin Resistance in MAFLD. Int. J. Mol. Sci..

[7] Bijnen M., Josefs T., Cuijpers I., Maalsen C.J., van de Gaar J., Vroomen M., Wijnands E., Rensen S.S., Greve J.W.M., Hofker M.H. (2018). Adipose tissue macrophages induce hepatic neutrophil recruitment and macrophage accumulation in mice. Gut.

[8] Bays H.E. (2011). Adiposopathy is “sick fat” a cardiovascular disease?. J. Am. Coll. Cardiol..

[9] Elton C.W., Tapscott E.B., Pories W.J., Dohm G.L. (1994). Effect of moderate obesity on glucose transport in human muscle. Horm. Metab. Res..

[10] Ciaraldi T.P., Ryan A.J., Mudaliar S.R., Henry R.R. (2016). Altered Myokine Secretion Is an Intrinsic Property of Skeletal Muscle in Type 2 Diabetes. PLoS ONE.

[11] Itoh N. (2014). FGF21 as a Hepatokine, Adipokine, and Myokine in Metabolism and Diseases. Front. Endocrinol..

[12] Moreno-Navarrete J.M., Ortega F., Serrano M., Guerra E., Pardo G., Tinahones F., Ricart W., Fernandez-Real J.M. (2013). Irisin is expressed and produced by human muscle and adipose tissue in association with obesity and insulin resistance. J. Clin. Endocrinol. Metab..

[13] Seldin M.M., Peterson J.M., Byerly M.S., Wei Z., Wong G.W. (2012). Myonectin (CTRP15), a novel myokine that links skeletal muscle to systemic lipid homeostasis. J. Biol. Chem..

[14] Hittel D.S., Berggren J.R., Shearer J., Boyle K., Houmard J.A. (2009). Increased secretion and expression of myostatin in skeletal muscle from extremely obese women. Diabetes.

[15] Trayhurn P., Wood I.S. (2004). Adipokines: Inflammation and the pleiotropic role of white adipose tissue. Br. J. Nutr..

[16] Cabre A., Lazaro I., Girona J., Manzanares J., Marimon F., Plana N., Heras M., Masana L. (2007). Retinol-binding protein 4 as a plasma biomarker of renal dysfunction and cardiovascular disease in type 2 diabetes. J. Intern. Med..

[17] Wentworth J.M., Naselli G., Brown W.A., Doyle L., Phipson B., Smyth G.K., Wabitsch M., O’Brien P.E., Harrison L.C. (2010). Pro-inflammatory CD11c+CD206+ adipose tissue macrophages are associated with insulin resistance in human obesity. Diabetes.

[18] O’Rourke R.W., Metcalf M.D., White A.E., Madala A., Winters B.R., Maizlin I.I., Jobe B.A., Roberts C.T., Slifka M.K., Marks D.L. (2009). Depot-specific differences in inflammatory mediators and a role for NK cells and IFN-gamma in inflammation in human adipose tissue. Int. J. Obes..

[19] Morris D.L., Cho K.W., Delproposto J.L., Oatmen K.E., Geletka L.M., Martinez-Santibanez G., Singer K., Lumeng C.N. (2013). Adipose Tissue Macrophages Function As Antigen-Presenting Cells and Regulate Adipose Tissue CD4+ T Cells in Mice. Diabetes.

[20] Elgazar-Carmon V., Rudich A., Hadad N., Levy R. (2008). Neutrophils transiently infiltrate intra-abdominal fat early in the course of high-fat feeding. J. Lipid Res..

[21] Rocha V.Z., Folco E.J., Sukhova G., Shimizu K., Gotsman I., Vernon A.H., Libby P. (2008). Interferon-gamma, a Th1 cytokine, regulates fat inflammation: A role for adaptive immunity in obesity. Circ. Res..

[22] Baron S. (1970). The Biological Significance of the Interferon System. JAMA Intern. Med..

[23] Isaacs A., Lindenmann J. (1957). Virus interference. I. The interferon. Proc. R. Soc. Lond. B Biol. Sci..

[24] Wheelock E.F. (1965). Interferon-Like Virus-Inhibitor Induced in Human Leukocytes by Phytohemagglutinin. Science.

[25] Kotenko S.V., Gallagher G., Baurin V.V., Lewis-Antes A., Shen M., Shah N.K., Langer J.A., Sheikh F., Dickensheets H., Donnelly R.P. (2003). IFN-lambdas mediate antiviral protection through a distinct class II cytokine receptor complex. Nat. Immunol..

[26] Cella M., Jarrossay D., Facchetti F., Alebardi O., Nakajima H., Lanzavecchia A., Colonna M. (1999). Plasmacytoid monocytes migrate to inflamed lymph nodes and produce large amounts of type I interferon. Nat. Med..

[27] Chehimi J., Starr S.E., Kawashima H., Miller D.S., Trinchieri G., Perussia B., Bandyopadhyay S. (1989). Dendritic cells and IFN-alpha-producing cells are two functionally distinct non-B, non-monocytic HLA-DR+ cell subsets in human peripheral blood. Immunology.

[28] Bell D.M., Roberts N.J., Hall C.B. (1983). Different antiviral spectra of human macrophage interferon activities. Nature.

[29] Lochhead R.B., Sonderegger F.L., Ma Y., Brewster J.E., Cornwall D., Maylor-Hagen H., Miller J.C., Zachary J.F., Weis J.H., Weis J.J. (2012). Endothelial cells and fibroblasts amplify the arthritogenic type I IFN response in murine Lyme disease and are major sources of chemokines in Borrelia burgdorferi-infected joint tissue. J. Immunol..

[30] Monroe K.M., McWhirter S.M., Vance R.E. (2010). Induction of type I interferons by bacteria. Cell Microbiol..

[31] Ferreyra G.A., Elinoff J.M., Demirkale C.Y., Starost M.F., Buckley M., Munson P.J., Krakauer T., Danner R.L. (2014). Late multiple organ surge in interferon-regulated target genes characterizes staphylococcal enterotoxin B lethality. PLoS ONE.

[32] Kirkwood J.M., Ernstoff M.S. (1990). Role of interferons in the therapy of melanoma. J. Investig. Dermatol..

[33] Fujioka N., Akazawa R., Sakamoto K., Ohashi K., Kurimoto M. (1995). Potential application of human interferon-alpha in microbial infections of the oral cavity. J. Interferon Cytokine Res..

[34] Spaapen R.M., Leung M.Y., Fuertes M.B., Kline J.P., Zhang L., Zheng Y., Fu Y.X., Luo X., Cohen K.S., Gajewski T.F. (2014). Therapeutic activity of high-dose intratumoral IFN-beta requires direct effect on the tumor vasculature. J. Immunol..

[35] Fuertes M.B., Kacha A.K., Kline J., Woo S.R., Kranz D.M., Murphy K.M., Gajewski T.F. (2011). Host type I IFN signals are required for antitumor CD8+ T cell responses through CD8{alpha}+ dendritic cells. J. Exp. Med..

[36] Diamond M.S., Kinder M., Matsushita H., Mashayekhi M., Dunn G.P., Archambault J.M., Lee H., Arthur C.D., White J.M., Kalinke U. (2011). Type I interferon is selectively required by dendritic cells for immune rejection of tumors. J. Exp. Med..

[37] Hooks J.J., Moutsopoulos H.M., Geis S.A., Stahl N.I., Decker J.L., Notkins A.L. (1979). Immune Interferon in the Circulation of Patients with Autoimmune Disease. N. Engl. J. Med..

[38] Higgs B.W., Liu Z., White B., Zhu W., White W.I., Morehouse C., Brohawn P., Kiener P.A., Richman L., Fiorentino D. (2011). Patients with systemic lupus erythematosus, myositis, rheumatoid arthritis and scleroderma share activation of a common type I interferon pathway. Ann. Rheum. Dis..

[39] Barrat F.J., Crow M.K., Ivashkiv L.B. (2019). Interferon target-gene expression and epigenomic signatures in health and disease. Nat. Immunol..

[40] Prummel M.F., Laurberg P. (2003). Interferon-alpha and autoimmune thyroid disease. Thyroid.

[41] Oritani K., Tomiyama Y. (2004). Interferon-zeta/limitin: Novel type I interferon that displays a narrow range of biological activity. Int. J. Hematol..

[42] Hardy M.P., Owczarek C.M., Jermiin L.S., Ejdeback M., Hertzog P.J. (2004). Characterization of the type I interferon locus and identification of novel genes. Genomics.

[43] Lukhele S., Boukhaled G.M., Brooks D.G. (2019). Type I interferon signaling, regulation and gene stimulation in chronic virus infection. Semin. Immunol..

[44] Ivashkiv L.B., Donlin L.T. (2014). Regulation of type I interferon responses. Nat. Rev. Immunol..

[45] Saleiro D., Platanias L.C. (2019). Interferon signaling in cancer. Non-canonical pathways and control of intracellular immune checkpoints. Semin. Immunol..

[46] Sica A., Mantovani A. (2012). Macrophage plasticity and polarization: In vivo veritas. J. Clin. Investig..

[47] Stetson D.B., Mohrs M., Reinhardt R.L., Baron J.L., Wang Z.E., Gapin L., Kronenberg M., Locksley R.M. (2003). Constitutive cytokine mRNAs mark natural killer (NK) and NK T cells poised for rapid effector function. J. Exp. Med..

[48] Schoenborn J.R., Wilson C.B. (2007). Regulation of Interferon-γ during Innate and Adaptive Immune Responses. Adv. Immunol..

[49] Lieberman L.A., Hunter C.A. (2002). The role of cytokines and their signaling pathways in the regulation of immunity to Toxoplasma gondii. Int. Rev. Immunol..

[50] Afkarian M., Sedy J.R., Yang J., Jacobson N.G., Cereb N., Yang S.Y., Murphy T.L., Murphy K.M. (2002). T-bet is a STAT1-induced regulator of IL-12R expression in naive CD4+ T cells. Nat. Immunol..

[51] Strengell M., Matikainen S., Siren J., Lehtonen A., Foster D., Julkunen I., Sareneva T. (2003). IL-21 in synergy with IL-15 or IL-18 enhances IFN-gamma production in human NK and T cells. J. Immunol..

[52] Fujii H., Ogasawara K., Otsuka H., Suzuki M., Yamamura K., Yokochi T., Miyazaki T., Suzuki H., Mak T.W., Taki S. (1998). Functional dissection of the cytoplasmic subregions of the IL-2 receptor betac chain in primary lymphocyte populations. EMBO J..

[53] Nagarajan N.A., Kronenberg M. (2007). Invariant NKT cells amplify the innate immune response to lipopolysaccharide. J. Immunol..

[54] Sica A., Bronte V. (2007). Altered macrophage differentiation and immune dysfunction in tumor development. J. Clin. Investig..

[55] Pan J., Zhang M., Wang J., Wang Q., Xia D., Sun W., Zhang L., Yu H., Liu Y., Cao X. (2004). Interferon-gamma is an autocrine mediator for dendritic cell maturation. Immunol. Lett..

[56] Snapper C.M., Paul W.E. (1987). Interferon-gamma and B cell stimulatory factor-1 reciprocally regulate Ig isotype production. Science.

[57] Okamura H., Tsutsi H., Komatsu T., Yutsudo M., Hakura A., Tanimoto T., Torigoe K., Okura T., Nukada Y., Hattori K. (1995). Cloning of a new cytokine that induces IFN-gamma production by T cells. Nature.

[58] Kane L.P., Lin J., Weiss A. (2000). Signal transduction by the TCR for antigen. Curr. Opin. Immunol..

[59] Yang J., Murphy T.L., Ouyang W., Murphy K.M. (1999). Induction of interferon-gamma production in Th1 CD4+ T cells: Evidence for two distinct pathways for promoter activation. Eur. J. Immunol..

[60] Darnell J.E., Kerr I.M., Stark G.R. (1994). Jak-STAT pathways and transcriptional activation in response to IFNs and other extracellular signaling proteins. Science.

[61] Sheppard P., Kindsvogel W., Xu W., Henderson K., Schlutsmeyer S., Whitmore T.E., Kuestner R., Garrigues U., Birks C., Roraback J. (2003). IL-28, IL-29 and their class II cytokine receptor IL-28R. Nat. Immunol..

[62] Prokunina-Olsson L., Muchmore B., Tang W., Pfeiffer R.M., Park H., Dickensheets H., Hergott D., Porter-Gill P., Mumy A., Kohaar I. (2013). A variant upstream of IFNL3 (IL28B) creating a new interferon gene IFNL4 is associated with impaired clearance of hepatitis C virus. Nat. Genet..

[63] Griffiths S.J., Dunnigan C.M., Russell C.D., Haas J.G. (2015). The Role of Interferon-lambda Locus Polymorphisms in Hepatitis C and Other Infectious Diseases. J. Innate Immun..

[64] Ank N., West H., Bartholdy C., Eriksson K., Thomsen A.R., Paludan S.R. (2006). Lambda interferon (IFN-lambda), a type III IFN, is induced by viruses and IFNs and displays potent antiviral activity against select virus infections in vivo. J. Virol..

[65] Zhang S.Y., Jouanguy E., Ugolini S., Smahi A., Elain G., Romero P., Segal D., Sancho-Shimizu V., Lorenzo L., Puel A. (2007). TLR3 Deficiency in Patients with Herpes Simplex Encephalitis. Science.

[66] Witte K., Gruetz G., Volk H.D., Looman A.C., Asadullah K., Sterry W., Sabat R., Wolk K. (2009). Despite IFN-lambda receptor expression, blood immune cells, but not keratinocytes or melanocytes, have an impaired response to type III interferons: Implications for therapeutic applications of these cytokines. Genes Immun..

[67] Doyle S.E., Schreckhise H., Khuu-Duong K., Henderson K., Rosler R., Storey H., Yao L., Liu H., Barahmand-pour F., Sivakumar P. (2006). Interleukin-29 uses a type 1 interferon-like program to promote antiviral responses in human hepatocytes. Hepatology.

[68] Hermant P., Demarez C., Mahlakoiv T., Staeheli P., Meuleman P., Michiels T. (2014). Human but not mouse hepatocytes respond to interferon-lambda in vivo. PLoS ONE.

[69] De Groen R.A., Groothuismink Z.M., Liu B.S., Boonstra A. (2015). IFN-lambda is able to augment TLR-mediated activation and subsequent function of primary human B cells. J. Leukoc. Biol..

[70] Yin Z., Dai J., Deng J., Sheikh F., Natalia M., Shih T., Lewis-Antes A., Amrute S.B., Garrigues U., Doyle S. (2012). Type III IFNs are produced by and stimulate human plasmacytoid dendritic cells. J. Immunol..

[71] Liu B.S., Janssen H.L., Boonstra A. (2011). IL-29 and IFNalpha differ in their ability to modulate IL-12 production by TLR-activated human macrophages and exhibit differential regulation of the IFNgamma receptor expression. Blood.

[72] Liu M.Q., Zhou D.J., Wang X., Zhou W., Ye L., Li J.L., Wang Y.Z., Ho W.Z. (2012). IFN-lambda3 inhibits HIV infection of macrophages through the JAK-STAT pathway. PLoS ONE.

[73] Su Q.J., Wang X., Zhou R.H., Guo L., Liu H., Li J.L., Ho W.Z. (2018). IFN-lambda4 inhibits HIV infection of macrophages through signalling of IFN-lambdaR1/IL-10R2 receptor complex. Scand. J. Immunol..

[74] Mahlakoiv T., Hernandez P., Gronke K., Diefenbach A., Staeheli P. (2015). Leukocyte-derived IFN-alpha/beta and epithelial IFN-lambda constitute a compartmentalized mucosal defense system that restricts enteric virus infections. PLoS Pathog..

[75] Lin J.D., Feng N., Sen A., Balan M., Tseng H.C., McElrath C., Smirnov S.V., Peng J., Yasukawa L.L., Durbin R.K. (2016). Distinct Roles of Type I and Type III Interferons in Intestinal Immunity to Homologous and Heterologous Rotavirus Infections. PLoS Pathog..

[76] Ingle H., Lee S., Ai T., Orvedahl A., Rodgers R., Zhao G., Sullender M., Peterson S.T., Locke M., Liu T.C. (2019). Viral complementation of immunodeficiency confers protection against enteric pathogens via interferon-lambda. Nat. Microbiol..

[77] Good C., Wells A.I., Coyne C.B. (2019). Type III interferon signaling restricts enterovirus 71 infection of goblet cells. Sci. Adv..

[78] Klinkhammer J., Schnepf D., Ye L., Schwaderlapp M., Gad H.H., Hartmann R., Garcin D., Mahlakoiv T., Staeheli P. (2018). IFN-lambda prevents influenza virus spread from the upper airways to the lungs and limits virus transmission. Elife.

[79] Ank N., Iversen M.B., Bartholdy C., Staeheli P., Hartmann R., Jensen U.B., Dagnaes-Hansen F., Thomsen A.R., Chen Z., Haugen H. (2008). An important role for type III interferon (IFN-lambda/IL-28) in TLR-induced antiviral activity. J. Immunol..

[80] Ye L., Schnepf D., Becker J., Ebert K., Tanriver Y., Bernasconi V., Gad H.H., Hartmann R., Lycke N., Staeheli P. (2019). Interferon-lambda enhances adaptive mucosal immunity by boosting release of thymic stromal lymphopoietin. Nat. Immunol..

[81] Stark G.R., Darnell J.E. (2012). The JAK-STAT pathway at twenty. Immunity.

[82] Teran-Cabanillas E., Montalvo-Corral M., Caire-Juvera G., Moya-Camarena S.Y., Hernandez J. (2013). Decreased interferon-alpha and interferon-beta production in obesity and expression of suppressor of cytokine signaling. Nutrition.

[83] Naghizadeh M., Baradaran B., Saghafi-Asl M., Amiri P., Shanehbandi D., Karamzad N., Mohamed-Khosroshahi L. (2018). Toll-like receptor signaling and serum levels of interferon beta and lipopolysaccharide binding protein are related to abdominal obesity: A case-control study between metabolically healthy and metabolically unhealthy obese individuals. Nutr. Res..

[84] Kintscher U., Hartge M., Hess K., Foryst-Ludwig A., Clemenz M., Wabitsch M., Fischer-Posovszky P., Barth T.F., Dragun D., Skurk T. (2008). T-lymphocyte infiltration in visceral adipose tissue: A primary event in adipose tissue inflammation and the development of obesity-mediated insulin resistance. Arterioscler. Thromb. Vasc. Biol..

[85] Satomura A., Oikawa Y., Haisa A., Suzuki S., Nakanishi S., Katsuki T., Shimada A. (2022). Clinical Significance of Insulin Peptide-specific Interferon-gamma-related Immune Responses in Ketosis-prone Type 2 Diabetes. J. Clin. Endocrinol. Metab..

[86] Ghazarian M., Revelo X.S., Nohr M.K., Luck H., Zeng K., Lei H., Tsai S., Schroer S.A., Park Y.J., Chng M.H.Y. (2017). Type I Interferon Responses Drive Intrahepatic T cells to Promote Metabolic Syndrome. Sci. Immunol..

[87] Bai J., Liu F. (2019). The cGAS-cGAMP-STING Pathway: A Molecular Link Between Immunity and Metabolism. Diabetes.

[88] Gaur P., Riehn M., Zha L., Koster M., Hauser H., Wirth D. (2021). Defective interferon amplification and impaired host responses against influenza virus in obese mice. Obesity.

[89] Muskiet F.A.J., Carrera-Bastos P., Pruimboom L., Lucia A., Furman D. (2022). Obesity and Leptin Resistance in the Regulation of the Type I Interferon Early Response and the Increased Risk for Severe COVID-19. Nutrients.

[90] Schmidt F.M., Weschenfelder J., Sander C., Minkwitz J., Thormann J., Chittka T., Mergl R., Kirkby K.C., Fasshauer M., Stumvoll M. (2015). Inflammatory cytokines in general and central obesity and modulating effects of physical activity. PLoS ONE.

[91] Yu H.R., Tain Y.L., Sheen J.M., Tiao M.M., Chen C.C., Kuo H.C., Hung P.L., Hsieh K.S., Huang L.T. (2016). Prenatal Dexamethasone and Postnatal High-Fat Diet Decrease Interferon Gamma Production through an Age-Dependent Histone Modification in Male Sprague-Dawley Rats. Int. J. Mol. Sci..

[92] Pacifico L., Di Renzo L., Anania C., Osborn J.F., Ippoliti F., Schiavo E., Chiesa C. (2006). Increased T-helper interferon-gamma-secreting cells in obese children. Eur. J. Endocrinol..

[93] Strissel K.J., DeFuria J., Shaul M.E., Bennett G., Greenberg A.S., Obin M.S. (2010). T-cell recruitment and Th1 polarization in adipose tissue during diet-induced obesity in C57BL/6 mice. Obesity.

[94] Dai G., Tan Y., Liu J., Yuan B., Song Q., Liu J., He S. (2020). The significance of IL-28B and CK-18 M30 levels in the diagnosis of non-alcoholic steatohepatitis in SD rats. Pathol. Res. Pract..

[95] Lin T.Y., Chiu C.J., Kuan C.H., Chen F.H., Shen Y.C., Wu C.H., Hsu Y.H. (2020). IL-29 promoted obesity-induced inflammation and insulin resistance. Cell. Mol. Immunol..

[96] Zhang H., Song B., He S. (2018). Interleukin 29 activates expression of tissue inhibitor of metalloproteinase 1 in macrophages via tolllike receptor 2. Mol. Med. Rep..

[97] Kolaczynski J.W., Taskinen M.R., Hilden H., Kiviluoto T., Cantell K., Koivisto V.A. (1992). Effects of interferon alpha on insulin binding and glucose transport in human adipocytes. Eur. J. Clin. Investig..

[98] Li C., Wang G., Sivasami P., Ramirez R.N., Zhang Y., Benoist C., Mathis D. (2021). Interferon-alpha-producing plasmacytoid dendritic cells drive the loss of adipose tissue regulatory T cells during obesity. Cell Metab..

[99] Samuel C.E. (2001). Antiviral actions of interferons. Clin. Microbiol. Rev..

[100] Ueki K., Kondo T., Kahn C.R. (2004). Suppressor of cytokine signaling 1 (SOCS-1) and SOCS-3 cause insulin resistance through inhibition of tyrosine phosphorylation of insulin receptor substrate proteins by discrete mechanisms. Mol. Cell. Biol..

[101] Wada T., Hoshino M., Kimura Y., Ojima M., Nakano T., Koya D., Tsuneki H., Sasaoka T. (2011). Both type I and II IFN induce insulin resistance by inducing different isoforms of SOCS expression in 3T3-L1 adipocytes. Am. J. Physiol. Endocrinol. Metab..

[102] Pan H., Yan B.S., Rojas M., Shebzukhov Y.V., Zhou H., Kobzik L., Higgins D.E., Daly M.J., Bloom B.R., Kramnik I. (2005). Ipr1 gene mediates innate immunity to tuberculosis. Nature.

[103] Ghosh A.R., Bhattacharya R., Bhattacharya S., Nargis T., Rahaman O., Duttagupta P., Raychaudhuri D., Liu C.S.C., Roy S., Ghosh P. (2016). Adipose Recruitment and Activation of Plasmacytoid Dendritic Cells Fuel Metaflammation. Immunol. Transplant..

[104] Koivisto V.A., Pelkonen R., Cantell K. (1989). Effect of interferon on glucose tolerance and insulin sensitivity. Diabetes.

[105] Newby B.N., Mathews C.E. (2017). Type I Interferon Is a Catastrophic Feature of the Diabetic Islet Microenvironment. Front. Endocrinol..

[106] Lombardi A., Tomer Y. (2017). Interferon alpha impairs insulin production in human beta cells via endoplasmic reticulum stress. J. Autoimmun..

[107] Reinehr T., Roth C.L. (2018). Inflammation Markers in Type 2 Diabetes and the Metabolic Syndrome in the Pediatric Population. Curr. Diab. Rep..

[108] Surendar J., Frohberger S.J., Karunakaran I., Schmitt V., Stamminger W., Neumann A.L., Wilhelm C., Hoerauf A., Hubner M.P. (2019). Adiponectin Limits IFN-gamma and IL-17 Producing CD4 T Cells in Obesity by Restraining Cell Intrinsic Glycolysis. Front. Immunol..

[109] Vandanmagsar B., Youm Y.H., Ravussin A., Galgani J.E., Stadler K., Mynatt R.L., Ravussin E., Stephens J.M., Dixit V.D. (2011). The NLRP3 inflammasome instigates obesity-induced inflammation and insulin resistance. Nat. Med..

[110] Chang C.C., Wu C.L., Su W.W., Shih K.L., Tarng D.C., Chou C.T., Chen T.Y., Kor C.T., Wu H.M. (2015). Interferon gamma-induced protein 10 is associated with insulin resistance and incident diabetes in patients with nonalcoholic fatty liver disease. Sci. Rep..

[111] Dunger A., Cunningham J.M., Delaney C.A., Lowe J.E., Green M.H., Bone A.J., Green I.C. (1996). Tumor necrosis factor-alpha and interferon-gamma inhibit insulin secretion and cause DNA damage in unweaned-rat islets. Extent of nitric oxide involvement. Diabetes.

[112] Sarvetnick N., Liggitt D., Pitts S.L., Hansen S.E., Stewart T.A. (1988). Insulin-dependent diabetes mellitus induced in transgenic mice by ectopic expression of class II MHC and interferon-gamma. Cell.

[113] Muir A., Peck A., Clare-Salzler M., Song Y.H., Cornelius J., Luchetta R., Krischer J., Maclaren N. (1995). Insulin immunization of nonobese diabetic mice induces a protective insulitis characterized by diminished intraislet interferon-gamma transcription. J. Clin. Investig..

[114] Khazen W., Distel E., Collinet M., Chaves V.E., M’Bika J.P., Chany C., Achour A., Benelli C., Forest C. (2007). Acute and selective inhibition of adipocyte glyceroneogenesis and cytosolic phosphoenolpyruvate carboxykinase by interferon gamma. Endocrinology.

[115] McGillicuddy F.C., Chiquoine E.H., Hinkle C.C., Kim R.J., Shah R., Roche H.M., Smyth E.M., Reilly M.P. (2009). Interferon gamma attenuates insulin signaling, lipid storage, and differentiation in human adipocytes via activation of the JAK/STAT pathway. J. Biol. Chem..

[116] Satoh M., Hoshino M., Fujita K., Iizuka M., Fujii S., Clingan C.S., Van Kaer L., Iwabuchi K. (2016). Adipocyte-specific CD1d-deficiency mitigates diet-induced obesity and insulin resistance in mice. Sci. Rep..

[117] Wentworth J.M., Zhang J.G., Bandala-Sanchez E., Naselli G., Liu R., Ritchie M., Smyth G.K., O’Brien P.E., Harrison L.C. (2017). Interferon-gamma released from omental adipose tissue of insulin-resistant humans alters adipocyte phenotype and impairs response to insulin and adiponectin release. Int. J. Obes..

[118] Keay S., Grossberg S.E. (1980). Interferon inhibits the conversion of 3T3-L1 mouse fibroblasts into adipocytes. Proc. Natl. Acad. Sci. USA.

[119] Sestan M., Marinovic S., Kavazovic I., Cekinovic D., Wueest S., Turk Wensveen T., Brizic I., Jonjic S., Konrad D., Wensveen F.M. (2018). Virus-Induced Interferon-gamma Causes Insulin Resistance in Skeletal Muscle and Derails Glycemic Control in Obesity. Immunity.

[120] Hannibal T.D., Schmidt-Christensen A., Nilsson J., Fransen-Pettersson N., Hansen L., Holmberg D. (2017). Deficiency in plasmacytoid dendritic cells and type I interferon signalling prevents diet-induced obesity and insulin resistance in mice. Diabetologia.

[121] Wu D., Sanin D.E., Everts B., Chen Q., Qiu J., Buck M.D., Patterson A., Smith A.M., Chang C.H., Liu Z. (2016). Type 1 Interferons Induce Changes in Core Metabolism that Are Critical for Immune Function. Immunity.

[122] Chan C.C., Damen M., Moreno-Fernandez M.E., Stankiewicz T.E., Cappelletti M., Alarcon P.C., Oates J.R., Doll J.R., Mukherjee R., Chen X. (2020). Type I interferon sensing unlocks dormant adipocyte inflammatory potential. Nat. Commun..

[123] Burke J.D., Platanias L.C., Fish E.N. (2014). Beta interferon regulation of glucose metabolism is PI3K/Akt dependent and important for antiviral activity against coxsackievirus B3. J. Virol..

[124] Alsaggar M., Mills M., Liu D. (2017). Interferon beta overexpression attenuates adipose tissue inflammation and high-fat diet-induced obesity and maintains glucose homeostasis. Gene Ther..

[125] Vlotides G., Sorensen A.S., Kopp F., Zitzmann K., Cengic N., Brand S., Zachoval R., Auernhammer C.J. (2004). SOCS-1 and SOCS-3 inhibit IFN-alpha-induced expression of the antiviral proteins 2,5-OAS and MxA. Biochem. Biophys. Res. Commun..

[126] Kuroda M., Nishiguchi M., Ugawa N., Ishikawa E., Kawabata Y., Okamoto S., Sasaki W., Miyatake Y., Sebe M., Masumoto S. (2020). Interferon regulatory factor 7 mediates obesity-associated MCP-1 transcription. PLoS ONE.

[127] Bai J., Cervantes C., Liu J., He S., Zhou H., Zhang B., Cai H., Yin D., Hu D., Li Z. (2017). DsbA-L prevents obesity-induced inflammation and insulin resistance by suppressing the mtDNA release-activated cGAS-cGAMP-STING pathway. Proc. Natl. Acad. Sci. USA.

[128] Wang X.L., Zhang L., Youker K., Zhang M.X., Wang J., LeMaire S.A., Coselli J.S., Shen Y.H. (2006). Free fatty acids inhibit insulin signaling-stimulated endothelial nitric oxide synthase activation through upregulating PTEN or inhibiting Akt kinase. Diabetes.

[129] Ishikawa H., Barber G.N. (2008). STING is an endoplasmic reticulum adaptor that facilitates innate immune signalling. Nature.

[130] Mao Y., Luo W., Zhang L., Wu W., Yuan L., Xu H., Song J., Fujiwara K., Abe J.I., LeMaire S.A. (2017). STING-IRF3 Triggers Endothelial Inflammation in Response to Free Fatty Acid-Induced Mitochondrial Damage in Diet-Induced Obesity. Arterioscler. Thromb. Vasc. Biol..

[131] Yang H., Youm Y.H., Vandanmagsar B., Ravussin A., Gimble J.M., Greenway F., Stephens J.M., Mynatt R.L., Dixit V.D. (2010). Obesity increases the production of proinflammatory mediators from adipose tissue T cells and compromises TCR repertoire diversity: Implications for systemic inflammation and insulin resistance. J. Immunol..

[132] Wong N., Fam B.C., Cempako G.R., Steinberg G.R., Walder K., Kay T.W., Proietto J., Andrikopoulos S. (2011). Deficiency in interferon-gamma results in reduced body weight and better glucose tolerance in mice. Endocrinology.

[133] McCabe K.M., Hsieh J., Thomas D.G., Molusky M.M., Tascau L., Feranil J.B., Qiang L., Ferrante A.W., Tall A.R. (2020). Antisense oligonucleotide treatment produces a type I interferon response that protects against diet-induced obesity. Mol. Metab..

[134] Zhang X.J., Zhang P., Li H. (2015). Interferon regulatory factor signalings in cardiometabolic diseases. Hypertension.

[135] Wang X.A., Zhang R., Jiang D., Deng W., Zhang S., Deng S., Zhong J., Wang T., Zhu L.H., Yang L. (2013). Interferon regulatory factor 9 protects against hepatic insulin resistance and steatosis in male mice. Hepatology.

[136] Wang X.A., Zhang R., Zhang S., Deng S., Jiang D., Zhong J., Yang L., Wang T., Hong S., Guo S. (2013). Interferon regulatory factor 7 deficiency prevents diet-induced obesity and insulin resistance. Am. J. Physiol. Endocrinol. Metab..

[137] Yang C.L., Sun F., Wang F.X., Rong S.J., Yue T.T., Luo J.H., Zhou Q., Wang C.Y., Liu S.W. (2022). The interferon regulatory factors, a double-edged sword, in the pathogenesis of type 1 diabetes. Cell Immunol..

[138] Donath M.Y. (2014). Targeting inflammation in the treatment of type 2 diabetes: Time to start. Nat. Rev. Drug Discov..

[139] Malozowski S., Sahlroot J.T. (2007). Interleukin-1–Receptor Antagonist in Type 2 Diabetes Mellitus. N. Engl. J. Med..

[140] Birk R.Z., Rubinstein M. (2006). IFN-alpha induces apoptosis of adipose tissue cells. Biochem. Biophys. Res. Commun..

[141] Lombardi A., Tsomos E., Hammerstad S.S., Tomer Y. (2018). Interferon alpha-The key trigger of type 1 diabetes. J. Autoimmun..

[142] Quiroga A.D., Comanzo C.G., Heit Barbini F.J., Lucci A., Vera M.C., Lorenzetti F., Ferretti A.C., Ceballos M.P., Alvarez M.L., Carrillo M.C. (2019). IFN-alpha-2b treatment protects against diet-induced obesity and alleviates non-alcoholic fatty liver disease in mice. Toxicol. Appl. Pharmacol..

[143] Ying W., Kanameni S., Chang C.A., Nair V., Safe S., Bazer F.W., Zhou B. (2014). Interferon tau alleviates obesity-induced adipose tissue inflammation and insulin resistance by regulating macrophage polarization. PLoS ONE.

[144] Campbell I.L., Kay T.W., Oxbrow L., Harrison L.C. (1991). Essential role for interferon-gamma and interleukin-6 in autoimmune insulin-dependent diabetes in NOD, Wehi mice. J. Clin. Investig..

[145] Sato T., Ikeda M., Yotsumoto S., Shimada Y., Higuchi T., Kobayashi H., Fukuda T., Ohashi T., Suda T., Ohteki T. (2013). Novel interferon-based pre-transplantation conditioning in the treatment of a congenital metabolic disorder. Blood.

